# Protein Biomarkers of Autism Spectrum Disorder Identified by Computational and Experimental Methods

**DOI:** 10.3389/fpsyt.2021.554621

**Published:** 2021-02-25

**Authors:** Fang Yao, Kaoyuan Zhang, Chengyun Feng, Yan Gao, Liming Shen, Xukun Liu, Jiazuan Ni

**Affiliations:** ^1^College of Life Science and Oceanography, Shenzhen University, Shenzhen, China; ^2^Department of Dermatology, Peking University Shenzhen Hospital, Shenzhen, China; ^3^Department of Child Healthcare, Maternal and Child Health Hospital of Baoan, Shenzhen, China

**Keywords:** autism spectrum disorder, blood, protein, biomarker, computational, experimental

## Abstract

**Background:** Autism spectrum disorder (ASD) is a complex neurodevelopmental disorder that affects millions of people worldwide. However, there are currently no reliable biomarkers for ASD diagnosis.

**Materials and Methods:** The strategy of computational prediction combined with experimental verification was used to identify blood protein biomarkers for ASD. First, brain tissue–based transcriptome data of ASD were collected from Gene Expression Omnibus database and analyzed to find ASD-related genes by bioinformatics method of significance analysis of microarrays. Then, a prediction program of blood-secretory proteins was applied on these genes to predict ASD-related proteins in blood. Furthermore, ELISA was used to verify these proteins in plasma samples of ASD patients.

**Results:** A total of 364 genes were identified differentially expressed in brain tissue of ASD, among which 59 genes were predicted to encode ASD-related blood-secretory proteins. After functional analysis and literature survey, six proteins were chosen for experimental verification and five were successfully validated. Receiver operating characteristic curve analyses showed that the area under the curve of SLC25A12, LIMK1, and RARS was larger than 0.85, indicating that they are more powerful in discriminating ASD cases from controls.

**Conclusion:** SLC25A12, LIMK1, and RARS might serve as new potential blood protein biomarkers for ASD. Our findings provide new insights into the pathogenesis and diagnosis of ASD.

## Introduction

Autism spectrum disorder (ASD) is a complex neurodevelopment disorder characterized by impairments in social interaction and communication, as well as expression of restricted interests and repetitive behavior (1). These symptoms would be presented during the first 3 years of life. Boys are with four to five times higher risk of autism than girls (2). According to reports of 2015, ~24.8 million people worldwide were affected by autism (3). In developed countries, the proportion of children with autism increased from 0.67% in 2000 to 1.5% in 2017 (4, 5). Obviously, the number of patients with ASD is increasing year by year.

Genetic and environmental factors are generally acknowledged as important contributors to the pathogenesis of ASD (6). However, the exact pathological mechanism remains uncertain and there are no effective treatments for ASD. Studies show that early intervention with behavioral therapy at an early stage can improve patient social communication and reduce anxiety and aggression. Thus, it is critical to detect ASD at an early stage (7, 8). Currently, the clinical diagnosis of ASD is based on the fifth edition of the Statistical Manual of Mental Disorder (DSM-V) (9), which may lead to exclusion of autistic individuals with mild form. Therefore, there is a need to find useful and reliable biomarkers to assist the diagnosis of autism.

Blood is a potential source for disease biomarker discovery because it contains large numbers of proteins associated with the physiology or pathology of disease. Several studies have been performed to search for blood biomarkers of ASD. Smith et al. (10) found that the combination of glutamine, glycine, and ornithine amino acid dysregulation identified a dysregulation in amino acid/branched-chain amino acid metabolism with good specificity and positive predictive value in the ASD subject cohort. Momeni et al. (11) found three differentially expressed peptides in the heparin plasma of children with ASD. Ngounou Wetie et al. (12) reported that apolipoproteins (Apos) ApoA1, ApoA4, and serum paraoxanase/arylesterase 1 (PON1) were increased in the sera of children with ASD. Wu et al. (13) proposed a movement biomarker to characterize the neurodevelopment level, which could differentiate ASD subjects from typically developing individuals. Howsmon et al. (14) developed multivariate statistical models to distinguish children with ASD from controls based on the metabolic abnormalities. Oztan et al. (15) employed a multidimensional neuropeptide analysis and found low blood neuropeptide receptor might act as promising biomarker of disease presence and symptom severity in ASD. Recently, we found a protein pattern that could distinguish the plasma samples of autistic children from healthy controls (16). In addition, we identified 41 proteins as differentially expressed proteins in the peripheral blood mononuclear cells of autistic children (17); three of them, i.e., complement C3 (C3), calreticulin (CALR), and alpha-1-antitrypsin (SERPINA1), are common differential proteins in the plasma (16). Despite these advances, there are still no diagnostic biomarkers available for ASD nowadays.

It is generally known that the blood–brain barrier (BBB) plays an important role in the defense of the central nervous system by limiting harmful solutes, macromolecules, and cells circulating from the bloodstream into the brain. However, several studies have shown that dysfunctions of BBB had a relationship with pathogenesis of neurological diseases including ASD (18–21), suggesting that some ASD-related proteins might be secreted from brain into blood as potential biomarkers. In addition, Cui et al. (22) developed a computational method to predict whether a protein could be secreted from tissue into blood with a high accuracy. Therefore, it would be possible to apply this program on the proteins encoded by the ASD-related genes to predict some potential ASD-related proteins in blood.

In this study, we identified blood protein biomarkers for ASD through computational prediction combined with experimental verification. First, we identified ASD-related genes by analyzing brain tissue–based gene expression data of autistic patients and healthy controls collected from a public database. Then, we predicted whether the protein products of these genes could be secreted into blood as ASD-related proteins. Further, we made bioinformatics analysis and literature survey on these proteins, and then selected some ASD-related proteins for verification in plasma of children with ASD by ELISA analysis.

## Materials and Methods

### Collection of Brain Tissue–Based Gene Expression Data of ASD Patients

**Figure 1:**
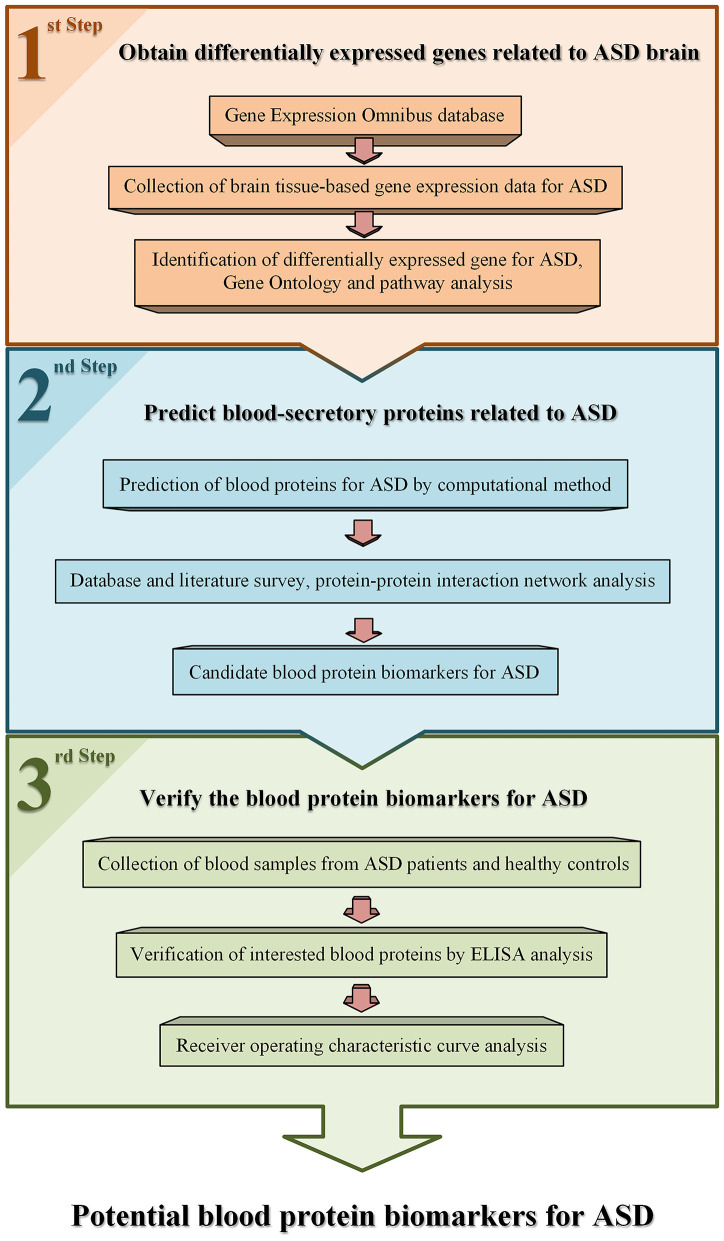
An overview of the work flow used in this study.

The workflow used in this study is shown in Figure 1. Gene expression data of brain tissues from patients with ASD and healthy controls were collected from public database Gene Expression Omnibus (GEO) (23). One dataset, GSE28521 (24), was selected for data analysis according to the following criteria. First, it contains both brain tissue samples of ASD patients and healthy controls. Second, the number of samples for ASD and controls are larger than 10, respectively. There are 79 brain tissue samples obtained from 19 autistic patients and 17 healthy controls in this dataset. Among these samples, 10, 16, and 13 samples are from cerebellum, frontal cortex, and temporal cortex of autism cases, and 11, 16, and 13 samples are from corresponding tissues of controls, respectively. The average age of the patients and controls were 24 (ranged from 2 to 56) and 34.6 (ranged from 16 to 56), respectively. The ratio of male to female was about 14:5 and 16:1 in the patients and controls. Detailed information of these samples in this dataset can be accessed from the GEO database.

### Identification of Differentially Expressed Genes for ASD

Generally, different tissues have different gene expression patterns. We investigated the differentially expressed genes of cerebellum, frontal cortex, and temporal cortex for ASD patients, respectively. A computational method, significance analysis of microarrays (SAM) (25), was employed to identify differentially expressed genes for ASD. A statistic delta was calculated for each gene in SAM, measuring how strong the relationship between gene expression and a response variable. R package “siggenes” was used to implement SAM analysis. To obtain the appropriate number of differentially expressed genes of ASD, delta was 1.2 and the false discovery rate (FDR) was 0.05 as cutoff.

To understand the functions of the differentially expressed genes, Database for Annotation, Visualization and Integrated Discovery (DAVID, http://david.abcc.ncifcrf.gov/) (26) was used to conduct Gene Ontology (GO) annotation and pathway analysis on these genes. In addition, functional interaction network analysis was performed using ClueGO cytoscape plugin [GlueGO v2.5.7; (27)].

### Prediction of ASD-Related Proteins in Blood

All differentially expressed genes of ASD were analyzed to determine whether their protein products could be secreted into blood by using a prediction program developed by Cui et al. (22). The main idea of the program is described as follows. Human proteins known to be blood secretory or not were collected from the published data to constitute the positive and negative training data, respectively. A list of protein features including sequence, structure, and chemical and physical properties was examined, and core features were selected according to their abilities in distinguishing the positive data from the negative. Based on the core features and training data, a prediction program for blood-secretory proteins was constructed by using support vector machine (SVM) (28) method.

In addition, to further determine whether these predicted proteins associated with ASD and presented in blood, we compared their genes with autism-associated gene database AutismKB (http://db.cbi.pku.edu.cn/autismkb_v2/) (29), and the proteins with plasma protein database (PPD, http://www.plasmaproteomedatabase.org/) (30), respectively. Moreover, protein–protein interaction (PPI) network analysis was conducted by using Lens for Enrichment and Network Studies of Proteins (LENS, http://severus.dbmi.pitt.edu/LENS/) (31) and Search Tool for the Retrieval of Interacting Genes/Proteins (String database, http://string-db.org/) (32).

### Verification of Potential Blood Protein Biomarkers for ASD by Using ELISA

After the aforementioned prediction of blood-secretory proteins associated with ASD, we selected some potential protein biomarkers for ASD to validate according to the following criteria. First, we ranked these proteins according to the likelihood of protein secretion into blood derived from the prediction program. Then we compared their genes with autism-associated gene database AutismKB and the proteins with plasma protein database. Further, we made functional analysis and literature survey on these proteins. Based on this criterion, we selected six proteins for verification by ELISA. ELISA analysis was conducted on blood samples of children with ASD and healthy controls. The research protocol of this study was permitted by the Human Research Ethics Committee of Shenzhen University and performed in accordance with the ethical standards laid down in the 1964 Declaration of Helsinki and its later amendments. A total of 40 subjects were recruited from Maternal and Child Health Hospital of Baoan between September 2017 and September 2018, including 20 children with ASD, and 20 age- and gender-matched healthy controls. The written consents were obtained from the caregivers of the participating children before this experiment. These patients were all diagnosed by a child neuropsychiatrist based on the criteria defined in the DSM-V (33). The male to female ratio was 4:1. The average age was 4.7 for patients and 4.5 for controls. The control cases had no known neurological disorders. There were no significant differences in weight, height, or body mass index (BMI) between the autistic children and healthy subjects. Blood samples (5 ml) were collected with EDTA-coated plastic tubes in the morning and then centrifuged at 3,000 × *g* for 10 min at room temperature. The supernatants were divided into aliquots and stored at −80°C until further analysis.

For ELISA analysis, the protein concentration was measured by a commercial ELISA kit (Uscn Life Science, Wuhan, China) according to the manufacturer's instructions, and then normalized by the total protein concentration determined by bicinchoninic acid (BCA) protein assay kit (Beyotime, Jiangsu, China). G-test (34) was used to detect the outliers in the normalized data. GraphPad Prism 5 software (GraphPad Software, San Diego, California) was applied to make statistical analyses on the concentrations of protein in ASD patients vs. healthy controls by using *t*-test with *p*-value < 0.05 as cutoff.

## Results

### Identification of Differentially Expressed Genes in Brain Tissues of ASD

There were 283 probes (3 up-regulated, 280 down-regulated) and 142 probes (3 up-regulated and 139 down-regulated) identified differentially expressed with FDR <0.05 as cutoff in the frontal cortex and temporal cortex of ASD, respectively. There were no differentially expressed probes found in the cerebellum of ASD. After combining the up- and down-regulated probes identified in the frontal and temporal cortex of ASD, six probes (corresponding to six genes) and 373 probes (corresponding to 358 genes) were differentially up- and down-regulated in cortex of ASD, respectively (Supplementary Tables 1, 2).

**Figure 2:**
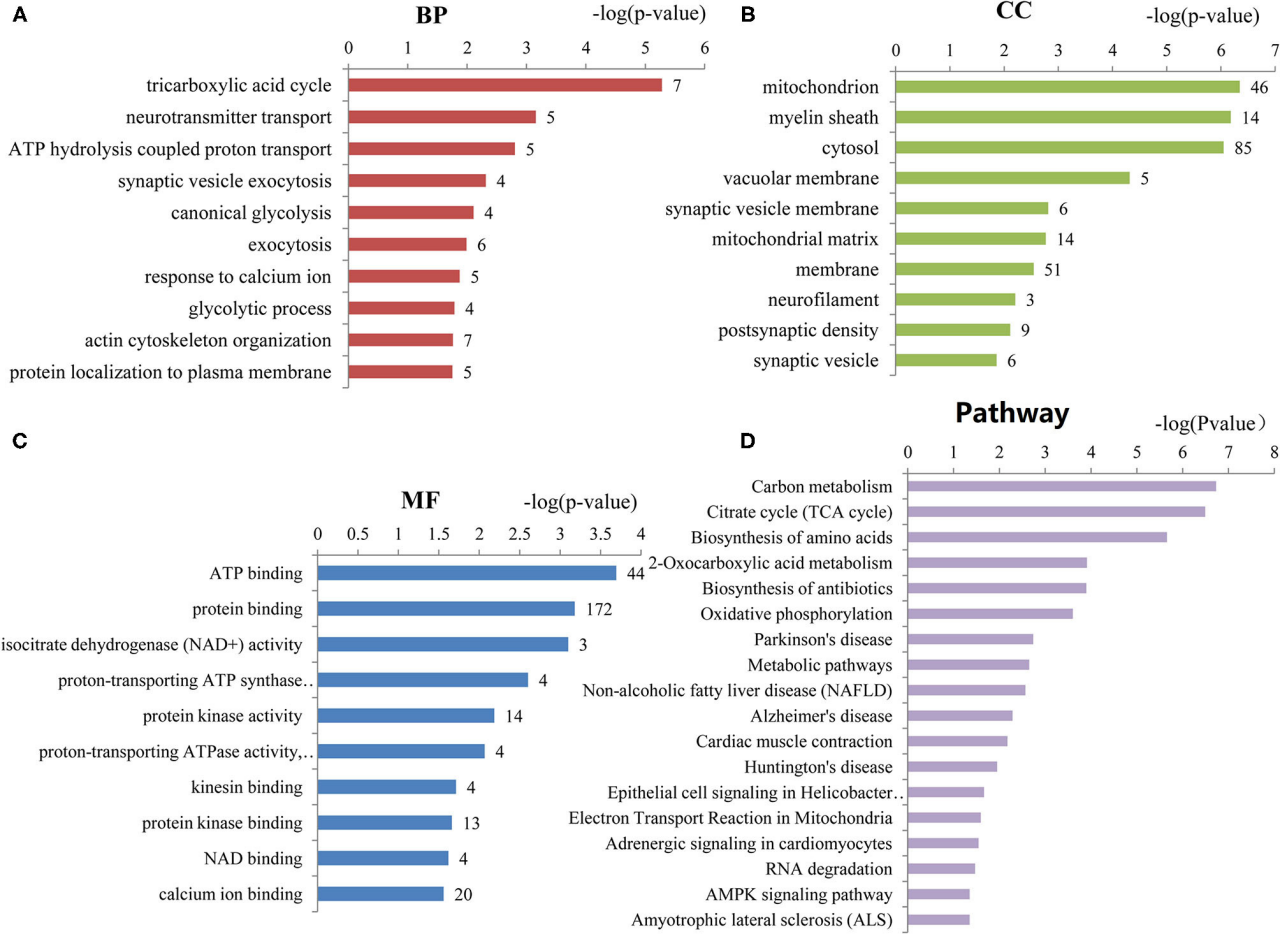
The top 10 enriched GO terms of biological processes, cellular components, and molecular functions. **(A)** Biological processes (BP) enriched by the differentially expressed genes of ASD. **(B)** Cellular components (CC) enriched by the differentially expressed genes of ASD. **(C)** Molecular functions (MF) enriched by the differentially expressed genes of ASD. The number of proteins associated with each category is presented at the end of each bar. **(D)** The pathways enriched by the differentially expressed genes of ASD. The number of proteins enriched in each pathway is at the end of each bar.

**Figure 3:**
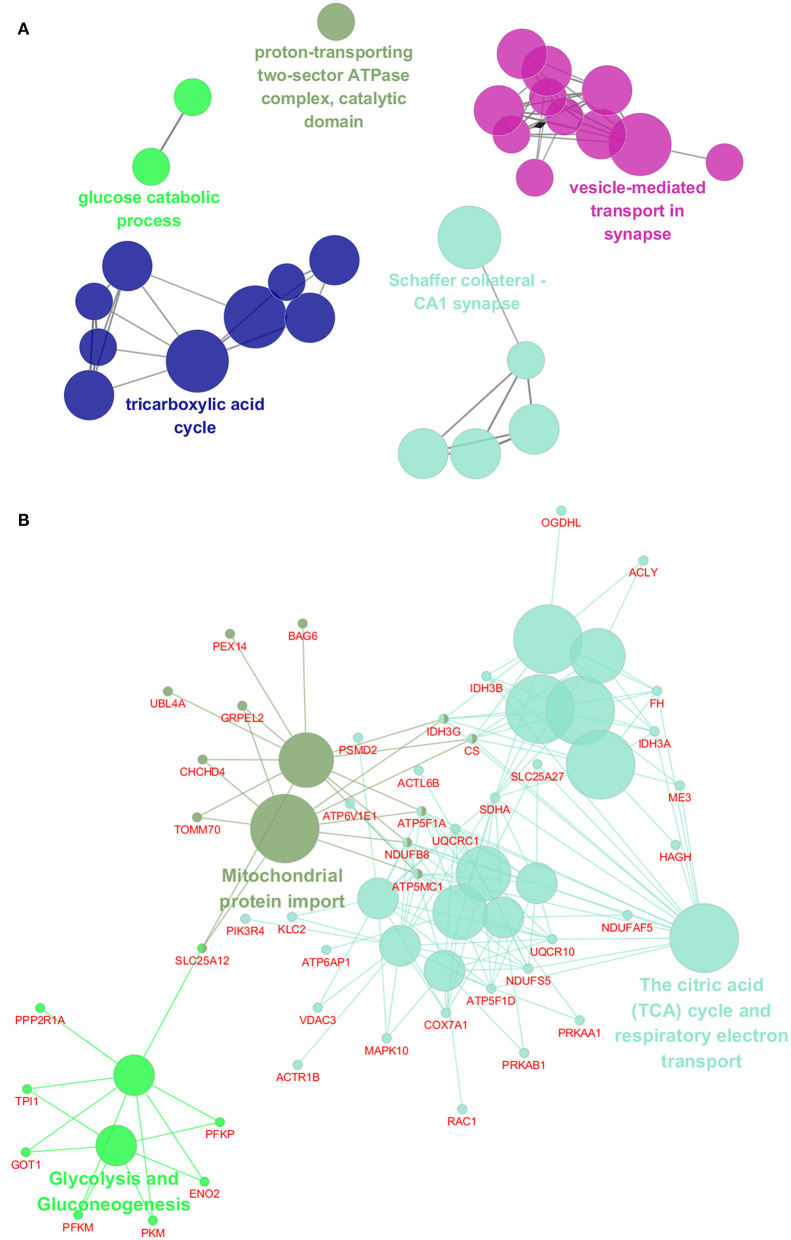
Functional interaction network analysis of the differentially expressed genes. **(A)** The differentially expressed genes were mapped to the GO categories, including biological processes and cellular components by using ClueGO cytoscape plugin. **(B)** The differentially expressed genes were mapped to the KEGG pathway, REACTOME pathway, and Wiki pathway by using ClueGO cytoscape plugin.

To assess the functions of these differentially expressed genes, GO annotation and pathway analyses were conducted by using DAVID database. A total of 72 GO terms and 18 pathways were significantly enriched by these genes with *p*-value <0.05 as threshold (Supplementary Tables 3–6). The enriched GO terms include 31 biological processes (BP), 19 cellular components (CC), and 22 molecular functions (MF). The top 10 enriched terms of BP, CC, and MF are shown in Figures 2A–C. BP analysis showed that they were involved in tricarboxylic acid (TCA) cycle, neurotransmitter transport, ATP hydrolysis coupled proton transport, synaptic vesicle exocytosis, canonical glycolysis, exocytosis, response to calcium ion, glycolytic process, actin and cytoskeleton organization, etc. CC analysis showed that they were associated with mitochondrion, myelin sheath, synaptic vesicle membrane, mitochondrial matrix, neurofilament, postsynaptic density, and synaptic vesicle. MF included ATP binding, calcium ion binding, syntaxin binding, etc. In addition, pathway analysis showed that some metabolic pathways were enriched, including carbon metabolism, TCA cycle, and oxidative phosphorylation (Figure 2D). Interestingly, pathways of Alzheimer's disease (AD), Huntington disease (HD), and amyotrophic lateral sclerosis (ALS) were also enriched by these genes. Similarly, the enrichment of BP and CC on these genes by ClueGO cytoscape plugin showed that they were mainly associated with glucose catabolic process, proton-transporting two-sector ATPase complex, catalytic domain, Schaffer collateral-CA1 synapse, and vesicle-mediated transport in synapse (Figure 3A, Supplementary Table 7). Pathway analysis showed that they were mainly involved in the citric acid (TCA) cycle and respiratory electron transport, mitochondrial protein import, and glycolysis and gluconeogenesis (Figure 3B, Supplementary Table 8).

### Prediction of ASD-Related Proteins in Blood

Based on the differentially expressed genes of ASD, we applied a computational program developed by Cui et al. (22) on these genes to predict whether their protein products could be secreted from tissue into blood. Subsequently, 59 proteins encoded by down-regulated genes were predicted to be blood-secretory proteins, suggesting that they might act as ASD-related proteins in blood (Supplementary Table 9).

**Figure 4:**
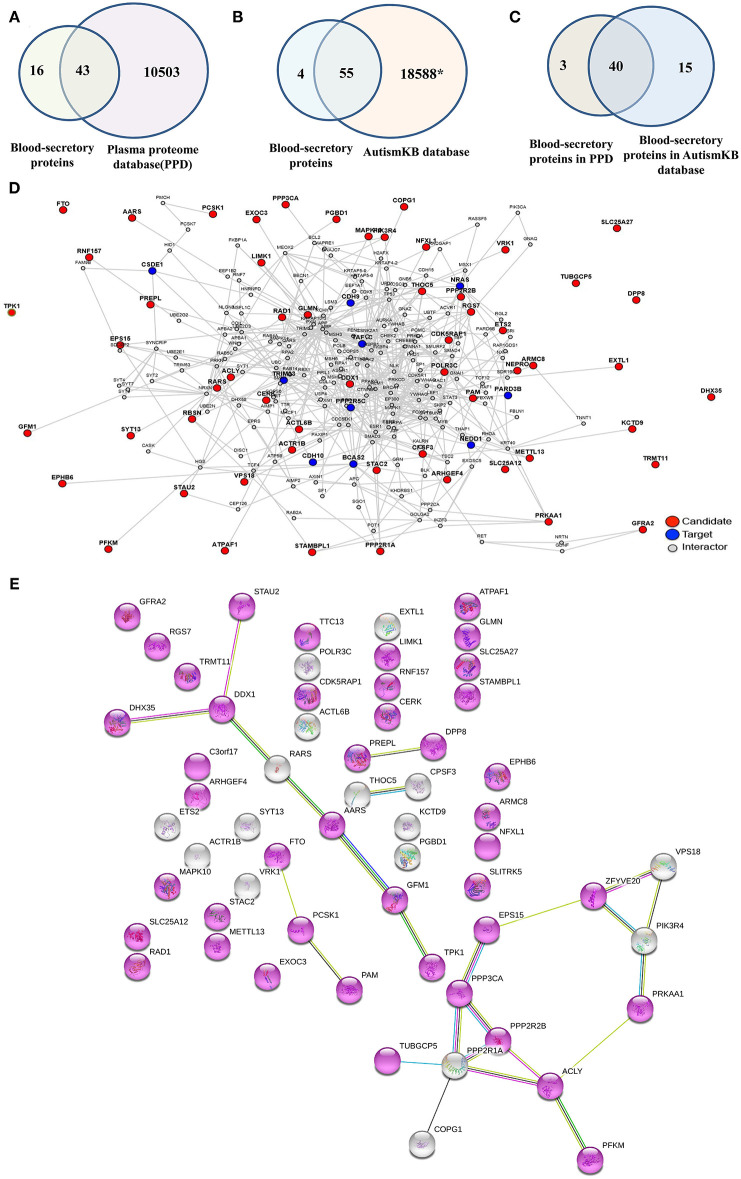
Database survey and protein–protein interaction network analysis on the 59 blood-secretory proteins. **(A)** Compared with plasma protein database. **(B)** Compared with ASD-related database (AutismKB). *The AutismKB database contains 1,379 genes, 5,420 copy number variants (CNVs)/structural variations (SVs), 11,669 single-nucleotide variations (SNVs)/insertions and deletions (InDels), and 172 linkage regions associated with ASD. **(C)** The blood-secretory proteins overlapped in plasma protein database and AutismKB database. **(D)** The protein–protein interaction network of these 59 blood-secretory proteins. The predicted blood-secretory proteins are shown as red nodes and autism pathology-related proteins are shown as blue nodes. **(E)** UniProt keywords were enriched by using String database. Gene with a node color of purplish red, whose Uniprot keyword is alternative splicing.

To examine whether the predicted blood-secretory proteins were present in blood, we compared these proteins with proteins in PPD (30) and found that 43 proteins were in common (Figure 4A, Supplementary Table 9). To further determine whether these predicted proteins were associated with ASD, we compared their corresponding genes with autism-related genes listed in the AutismKB (29) and found that 55 genes were reported associated with ASD except *TTC13, RARS, THOC5*, and *ATPAF1* (Figure 4B, Supplementary Table 9). There were 40 overlapping blood-secretory proteins between PPD and AutismKB database (Figure 4C, Supplementary Table 9). After literature survey on the four genes, we found that *RARS* and *THOC5* had been reported related to brain development (35, 36).

In order to understand how these predicted proteins were involved in the pathogenesis of ASD, we conducted a PPI network analysis on these proteins by using a web tool of LENS. Figure 4D shows the network, which was constructed by 59 blood-secretory proteins (red nodes) input as candidate proteins and 15 proteins known related to autism pathology (blue nodes) provided as targeted proteins. From the network, we found that most of these blood-secretory proteins were connected with the targeted proteins except SLITRK5, TPK1, SLC25A27, DHX35, DPP8, TTC13, TUBGCP5, FTO, and TRMT11. Interestingly, String database analysis showed that 43 proteins might be associated with alternative splicing (Figure 4E).

### Verification of the Potential Protein Biomarkers for ASD by ELISA

**Figure 5:**
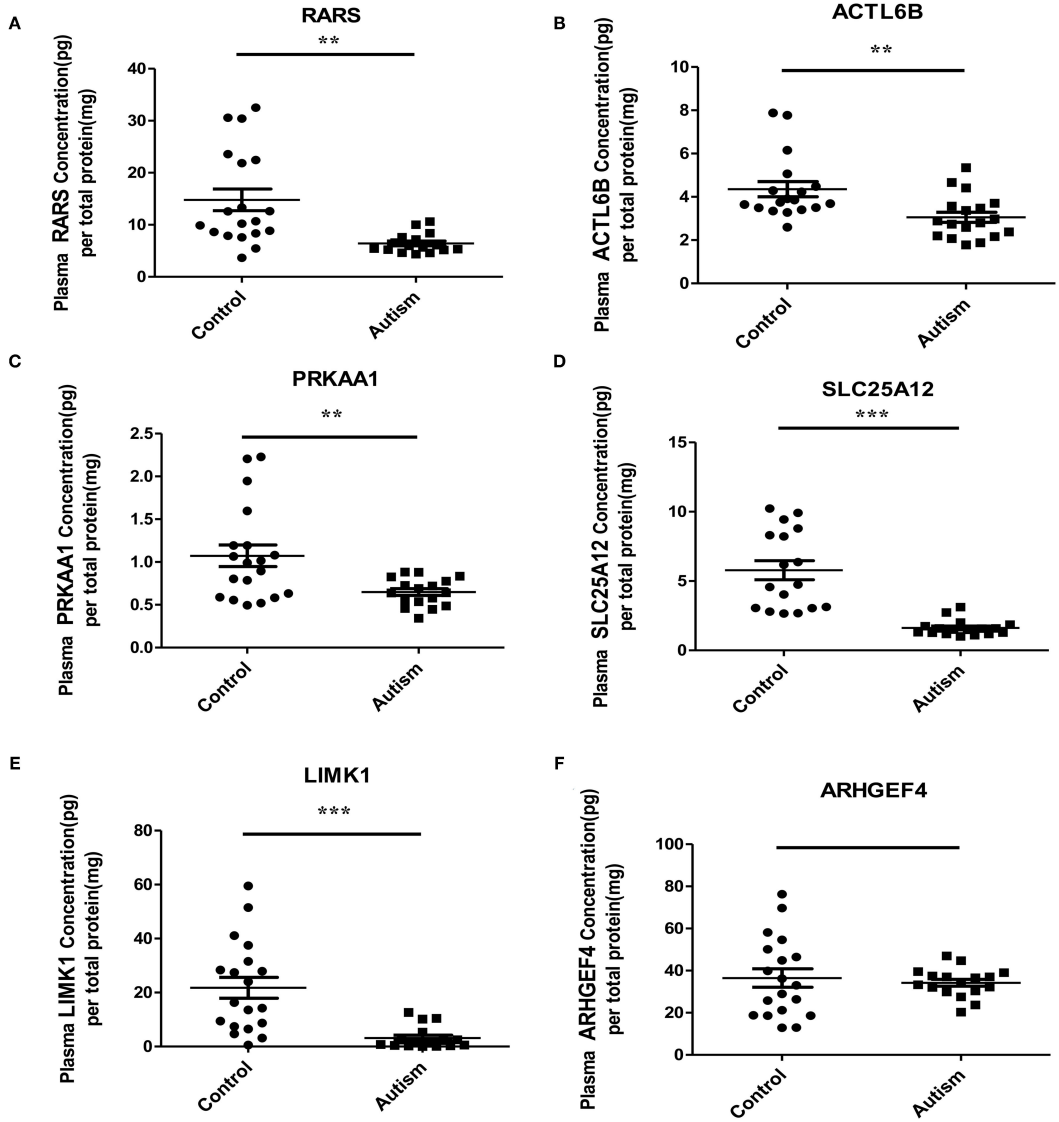
Verification of the potential blood protein biomarkers for ASD by ELISA. **p < 0.005; ***p < 0.0005.

Based on the possibilities of proteins secreting into blood and their functions, six proteins were selected for validation in blood samples of children with ASD and healthy controls, including arginine-tRNA ligase, cytoplasmic (RARS), actin-like protein 6B (ACTL6B), 5′-AMP-activated protein kinase catalytic subunit alpha-1 (PRKAA1), calcium-binding mitochondrial carrier protein Aralar1 (SLC25A12), LIM domain kinase 1 (LIMK1), and rho guanine nucleotide exchange factor 4 (ARHGEF4) (Table 1). As shown in Figure 5, five proteins, RARS, ACTL6B, PRKAA1, SLC25A12, and LIMK1, were significantly down-regulated in plasma samples of ASD, which were consistent with the expression changes of their corresponding genes mentioned previously. Even though the expression level of ARHGEF4 was not significantly down-regulated in autism samples, it was still expressed lower in autism samples compared with controls.

**Table 1:** Six predicted blood-secretory proteins selected for validation in this study.

**No**.	**Gene**	**UniProt ID**	**Protein^a^**	**Function^b^**
1	*RARS*	P54136	Arginine–tRNA ligase, cytoplasmic	Protein synthesis, inflammatory
2	*ACTL6B*	O94805	Actin-like protein 6B	Transcriptional activation and chromatin remodeling
3	*ARHGEF4*	Q9NR80	Rho guanine nucleotide exchange factor 4	High levels in the brain and is involved in cell migration and cell–cell adhesion
4	*PRKAA1*	Q13131	5′-AMP-activated protein kinase catalytic subunit alpha-1	Cellular energy metabolism, cell growth and proliferation, phosphorylation
5	*SLC25A12*	O75746	Calcium-binding mitochondrial carrier protein Aralar1	Calcium ion binding, amino acid metabolism
6	*LIMK1*	P53667	LIM domain kinase 1	Stimulates axonal outgrowth and may be involved in brain development

a *Except RARS did not match the two databases (i.e., AutismKB and plasma protein database), all the other proteins were matched. However, RARS has important functions and may be implied in the pathogenesis of ASD*.

b *Functional information obtained from UniProt database (https://www.uniprot.org/)*.

**Figure 6:**
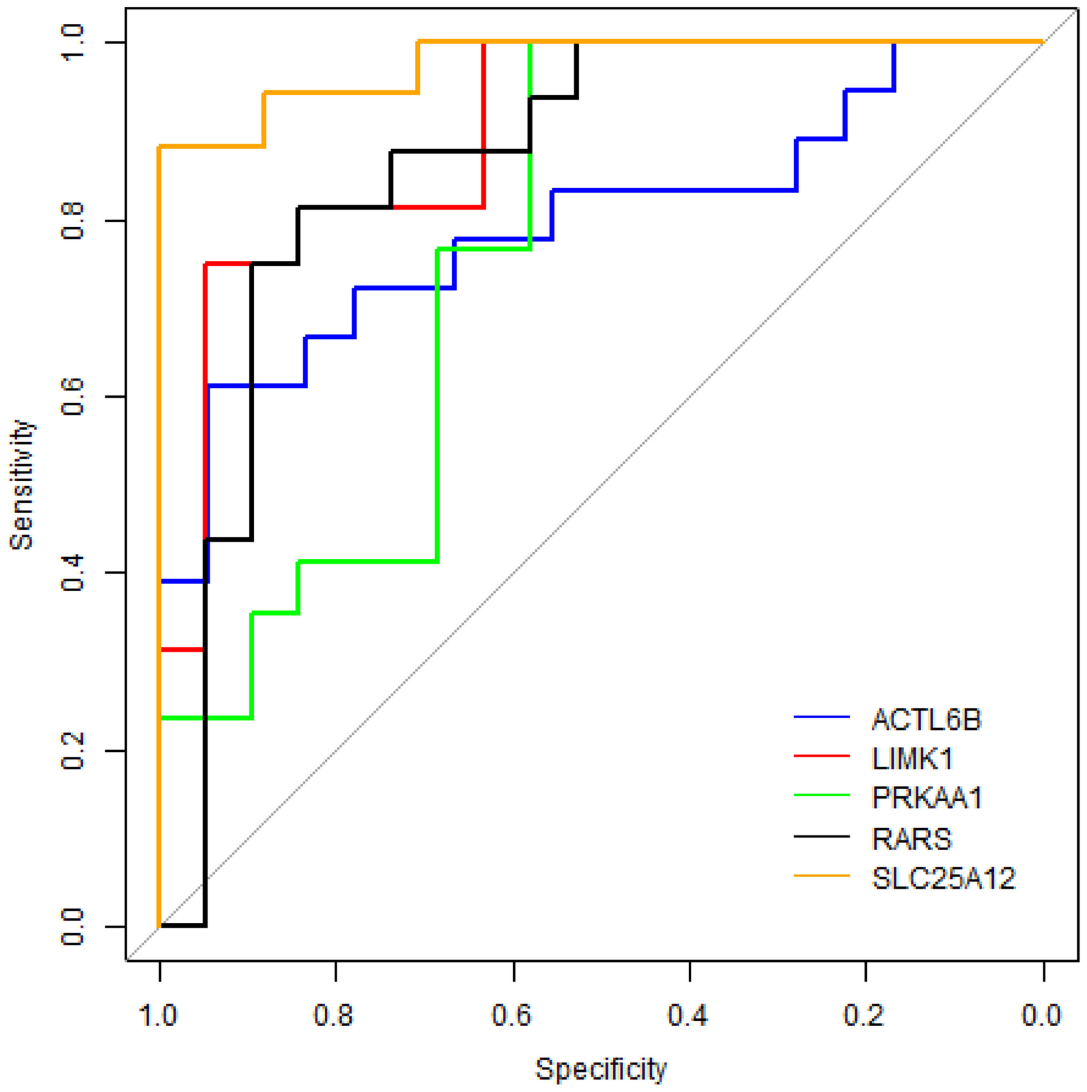
ROC curve analyses on five differentially expressed proteins in plasma samples of ASD patients. The blue line represents protein ACTL6B, the red line is LIMK1, the green line is PRKAA1, the black line is RARS, and the orange line is SLC25A12.

To evaluate the performance of these five proteins in distinguishing samples of ASD from healthy controls, receiver operating characteristic (ROC) curve analyses were carried out on protein concentrations measured by ELISA. Figure 6 shows that SLC25A12 has the most discriminative ability with the area under curve (AUC) of 0.976 (sensitivity 100%, specificity 88.2%), and the AUCs of LIMK1 and RARS are 0.898 (sensitivity 94.7%, specificity 75.0%) and 0.862 (sensitivity 84.2%, specificity 81.2%), respectively. The remaining two proteins ACTL6B and PRKAA1 are with AUCs of 0.793 and 0.768, respectively.

## Discussion

ASD is a neurodevelopment disorder that has affected the health of millions of people. However, the pathogenesis of ASD is poorly understood and there are no reliable diagnostic biomarkers currently. In this study, we identified the potential blood protein biomarkers for ASD by a new strategy of computational prediction in conjunction with experimental validation, which could provide a more effective and specific way for biomarker discovery in blood (37).

First of all, 364 differentially expressed genes were identified for ASD based on transcriptome analysis. Functional enrichment analysis showed that these genes were mainly involved in BPs of TCA cycle, neurotransmitter transport, and synaptic vesicle exocytosis; CCs of mitochondrion, myelin sheath, and synaptic vesicle membrane; actin and neurofilament cytoskeleton organization and synapse; MFs of ATP binding, calcium ion binding, and syntaxin binding; and pathways of metabolic, nervous system diseases, and alternative splicing, which are all known to be associated with the pathophysiology of ASD.

From the aforementioned functional analysis, it could be speculated that the mitochondria, myelin sheath, synapses, and cytoskeleton of neurofilaments are impaired in the brains of children with ASD. Previous studies have reported that mitochondrial dysfunction seemed to be the most prevalent metabolic disease associated with ASD (38, 39). Mitochondrial dysfunction could lead to metabolic changes. Here, the metabolic abnormalities include carbon metabolism, TCA cycle, oxidative phosphorylation, glycolysis, and gluconeogenesis, which are consistent with previous published research (40–42). In addition, changes in myelin sheath, and actin and neurofilament cytoskeleton have been reported associated with ASD (43–47). In agreement with previous research (24, 44, 45, 47–51), the genes associated with pre-synaptic and post-synaptic proteins, synaptic vesicles, and neurotransmitter transport were observed as significantly changed in ASD subjects vs. controls. Furthermore, it has been reported that differential alternative splicing was observed in ASD brains and blood (24, 52, 53), and the unfolded protein response and altered endoplasmic reticulum (ER) stress have also been reported to be associated with ASD (17, 54, 55). It should be a concern that these factors are interrelated with each other. Dysfunction of mitochondria might cause impairment of synaptic function, and both of them are related to neurological diseases such as AD, schizophrenia, and so on (42, 56). Alternative splicing has been reported to be related to the expression of synaptic-related genes in ASD (57). Mutations linked to ASDs in synaptic proteins such as NLGN3, CASPR2, and CADM1 might lead to ER stress conditions (58).

After functional analysis and literature survey, six proteins were selected for verified in plasma samples of ASD, and five were successfully verified, including RARS, ACTL6B, PRKAA1, SLC25A12, and LIMK1. Among them, RARS acts as an enzyme essential for RNA translation and plays an important role in myelination (35). *ACTL6B* was identified as a candidate risk gene for ASD with functions of neuron-specific chromatin remodeling and neurodevelopment (59, 60). PRKAA1, a catalytic subunit of protein kinase A (PKA), plays a key role in regulating cellular energy metabolism. It was found that regression in ASD might be associated with decreased PKA-mediated phosphorylation of proteins and abnormalities in cellular signaling (61). PRKAA1 has also been reported in several studies related to autism and/or ASD including linkage studies (62–64), NGS *de novo* mutation studies (65), and genome-wide association studies (66). *SLC25A12* has been proposed as a candidate gene for ASD due to its important role in mitochondrial function and ATP synthesis (67). Some research showed that single nucleotide polymorphism in *SLC25A12* might significantly contribute to ASD risk (68, 69). Increased evidence suggests that it may play a critical role in the pathogenesis of ASD (69–72). In particular, it has been reported that SLC25A12 is associated with autism of the Han Chinese in Taiwan (70). Meanwhile, it is worth noting that *SLC25A12* is with the highest “evidence score” in the AutismKB, indicating that it is closely associated with ASD. Moreover, *LIMK1* stimulates axonal outgrowth and involves in neurodevelopment and synaptic plasticity (73, 74). It has been reported to be related to ASD (24, 63). Furthermore, *ARHGEF* has been reported to be associated with copy number variants (CNVs) in children with ASD (75–77). Here, although it has no significant difference between the cases and the control group, the trend was in line with expectations and a larger sample size might be needed to verify the change.

ROC curve analyses showed that the AUCs of SLC25A12, LIMK1, and RARS were larger than 0.85, indicating that they are more powerful in distinguishing samples of ASD from healthy controls and might serve as new potential protein biomarkers for ASD in blood. As far as we know, this is the first study to investigate blood protein biomarkers for ASD through such a strategy. Proteins SLC25A12, LIMK1, and RARS were first reported here as new potential blood protein biomarkers for ASD. Clearly, these findings are needed to be confirmed on large number of samples.

In conclusion, the combination of computational prediction and experimental validation was used to identify blood protein biomarkers for ASD. A total of 364 differentially expressed genes were found in ASD, out of which 59 genes were predicted that their protein products could be secreted into blood as candidate ASD-related blood proteins. After functional analysis and literature survey, six proteins were selected for experimental validation and five were successfully verified in the plasma samples of ASD. ROC analysis showed that SLC25A12, LIMK1, and RARS are more powerful in differentiating ASD samples from controls and might serve as new potential protein biomarkers for ASD in blood.

## Data Availability Statement

The original contributions presented in the study are included in the article/Supplementary Material, further inquiries can be directed to the corresponding author.

## Ethics Statement

The studies involving human participants were reviewed and approved by the Human Research Ethics Committee of Shenzhen University. Written informed consent to participate in this study was provided by the participants' legal guardian/next of kin.

## Author Contributions

LS conceived and designed the study and revised the article. FY performed statistical analysis, data interpretation, and drafted the first article. KZ and XL contributed to experimental validation and data analysis. CF and YG contributed to acquisition of blood samples and performed correlation analysis. JN coordinated the study design and article revision. All authors contributed to the article and approved the submitted version.

## Conflict of Interest

The authors declare that the research was conducted in the absence of any commercial or financial relationships that could be construed as a potential conflict of interest.

## Abbreviations

ASD: autism spectrum disorder
BBB: blood–brain barrier
GEO: gene expression omnibus
SAM: significance analysis of microarrays
FDR: false discovery rate
DAVID, database for annotation: visualization and integrated discovery
GO: gene ontology
SVM: support vector machine
PPD: plasma protein database
PPI: protein–protein interaction
LENS: lens for enrichment and network studies of proteins
BP: biological process
CC: cellular component
MF: molecular function
HD: Huntington disease
ALS: amyotrophic lateral sclerosis
ROC: receiver operating characteristic
AUC: area under curve.
