# Supplementary material for: Protein Biomarkers of Autism Spectrum Disorder Identified by Computational and Experimental Methods
Source: Front Psychiatry. 2021 Feb 25;12:554621. doi: 10.3389/fpsyt.2021.554621 (PMC7947305; doi:10.3389/fpsyt.2021.554621)
Supplement: Supplementary file 1 [file Table_1.DOCX]

Supplementary Material

# Supplementary Tables

## Supplementary Table S1. The total up-regulated probes and their corresponding genes in ASD patients.

| **No.** | **Probes** | **Genes** |
| --- | --- | --- |
| 1 | ILMN_2365549 | *BRPF1* |
| 2 | ILMN_2133360 | *LOC91561* |
| 3 | ILMN_2385220 | *DFFA* |
| 4 | ILMN_1706990 | *ZNF271* |
| 5 | ILMN_1651496 | *HIST1H2BD* |
| 6 | ILMN_2123665 | *SBF2* |

## Supplementary Table S2. The total down-regulated probes and their corresponding genes in ASD patients.

| **No.** | **Probes** | **Genes** |
| --- | --- | --- |
| 1 | ILMN_1719392 | *FH* |
| 2 | ILMN_1789266 | *CCDC25* |
| 3 | ILMN_1763264 | *MRPL2* |
| 4 | ILMN_1728540 | *FUNDC1* |
| 5 | ILMN_1789166 | *SHD* |
| 6 | ILMN_1796235 | *CIRH1A* |
| 7 | ILMN_1788053 | *SLC25A12* |
| 8 | ILMN_1697585 | *ACTR6* |
| 9 | ILMN_1697286 | *SF3A1* |
| 10 | ILMN_1743303 | *TTC1* |
| 11 | ILMN_1659820 | *PTD004* |
| 12 | ILMN_1725620 | *ZNF403* |
| 13 | ILMN_1802628 | *PPAPDC2* |
| 14 | ILMN_1805737 | *PFKP* |
| 15 | ILMN_1811367 | *MAT2B* |
| 16 | ILMN_1665547 | *CADPS* |
| 17 | ILMN_1789186 | *OBFC1* |
| 18 | ILMN_1813938 | *CHCHD4* |
| 19 | ILMN_1741156 | *ARMCX5* |
| 20 | ILMN_1702205 | *PCDHA5* |
| 21 | ILMN_2393169 | *THOC5* |
| 22 | ILMN_2120210 | *DSCR1L1* |
| 23 | ILMN_1804629 | *TPK1* |
| 24 | ILMN_1795678 | *POLR3C* |
| 25 | ILMN_1791949 | *PGBD1* |
| 26 | ILMN_1698533 | *IDH3A* |
| 27 | ILMN_1699623 | *FAM81A* |
| 28 | ILMN_2054213 | *HSMPP8* |
| 29 | ILMN_1769637 | *RNMT* |
| 30 | ILMN_2312732 | *DPP8* |
| 31 | ILMN_1797950 | *EXTL2* |
| 32 | ILMN_1765044 | *CUTC* |
| 33 | ILMN_1785202 | *STAT4* |
| 34 | ILMN_1739397 | *GLMN* |
| 35 | ILMN_1707137 | *LOC400566* |
| 36 | ILMN_2165463 | *HAPLN4* |
| 37 | ILMN_1788538 | *NCALD* |
| 38 | ILMN_1654385 | *ASB13* |
| 39 | ILMN_1737462 | *OXR1* |
| 40 | ILMN_1683888 | *SRP72* |
| 41 | ILMN_1733667 | *DHX35* |
| 42 | ILMN_1698726 | *SLC25A27* |
| 43 | ILMN_2236800 | *SON* |
| 44 | ILMN_1754727 | *GPRASP2* |
| 45 | ILMN_1760335 | *ADPRHL1* |
| 46 | ILMN_1667883 | *THOC5* |
| 47 | ILMN_1708204 | *COPG* |
| 48 | ILMN_1682197 | *NFXL1* |
| 49 | ILMN_1783889 | *PRKAA1* |
| 50 | ILMN_1731354 | *PSARL* |
| 51 | ILMN_1671191 | *UQCRC1* |
| 52 | ILMN_1754076 | *CACNA2D3* |
| 53 | ILMN_1741204 | *KLHDC2* |
| 54 | ILMN_1722156 | *RWDD2* |
| 55 | ILMN_1747078 | *HYLS1* |
| 56 | ILMN_2081813 | *PCSK1* |
| 57 | ILMN_1658094 | *ZNF365* |
| 58 | ILMN_1724376 | *C2orf30* |
| 59 | ILMN_1751378 | *RARS* |
| 60 | ILMN_1769720 | *STAU2* |
| 61 | ILMN_1763460 | *NHP2L1* |
| 62 | ILMN_1657855 | *ACTL6B* |
| 63 | ILMN_1661491 | *SH3GL2* |
| 64 | ILMN_1654543 | *MED6* |
| 65 | ILMN_1808765 | *ZNF25* |
| 66 | ILMN_1664537 | *USP11* |
| 67 | ILMN_2392635 | *ABCF1* |
| 68 | ILMN_1773307 | *NAP1L5* |
| 69 | ILMN_1736015 | *PHF17* |
| 70 | ILMN_1662364 | *AARS* |
| 71 | ILMN_1803813 | *ASTE1* |
| 72 | ILMN_1691648 | *TRO* |
| 73 | ILMN_1683932 | *ZNF425* |
| 74 | ILMN_2205032 | *MAGEE1* |
| 75 | ILMN_1677919 | *GMPR2* |
| 76 | ILMN_1711023 | *CDK5RAP1* |
| 77 | ILMN_2134062 | *ALKBH3* |
| 78 | ILMN_1750092 | *SLA/LP* |
| 79 | ILMN_1759801 | *DPP8* |
| 80 | ILMN_1755222 | *C9orf82* |
| 81 | ILMN_1778425 | *DPP10* |
| 82 | ILMN_1739454 | *USP34* |
| 83 | ILMN_2365595 | *GMPR2* |
| 84 | ILMN_1789419 | *SEC6L1* |
| 85 | ILMN_1804610 | *NFX1* |
| 86 | ILMN_2365569 | *ICA1* |
| 87 | ILMN_1665357 | *EPS15* |
| 88 | ILMN_1721713 | *EXOSC9* |
| 89 | ILMN_2181125 | *NAPB* |
| 90 | ILMN_1803036 | *TARBP1* |
| 91 | ILMN_1712432 | *PSMD2* |
| 92 | ILMN_1805828 | *VRK1* |
| 93 | ILMN_1737611 | *VAMP1* |
| 94 | ILMN_2156267 | *EIF2AK1* |
| 95 | ILMN_1708983 | *CASC1* |
| 96 | ILMN_1777663 | *TOP2B* |
| 97 | ILMN_1758827 | *RTN4IP1* |
| 98 | ILMN_1697153 | *ZDHHC17* |
| 99 | ILMN_1684461 | *CADPS2* |
| 100 | ILMN_2218208 | *SPARCL1* |
| 101 | ILMN_1723626 | *APBA2* |
| 102 | ILMN_2331658 | *C3orf17* |
| 103 | ILMN_2313901 | *PAM* |
| 104 | ILMN_1718129 | *MAP2K5* |
| 105 | ILMN_1724811 | *PARN* |
| 106 | ILMN_2137066 | *ZNF7* |
| 107 | ILMN_1800270 | *GABRG2* |
| 108 | ILMN_2151114 | *VSNL1* |
| 109 | ILMN_1803045 | *TUBGCP5* |
| 110 | ILMN_1658499 | *SYT13* |
| 111 | ILMN_1709728 | *SLC30A5* |
| 112 | ILMN_2343047 | *ABCB9* |
| 113 | ILMN_1748281 | *MAPK10* |
| 114 | ILMN_2202481 | *UBLCP1* |
| 115 | ILMN_1659937 | *ZBTB24* |
| 116 | ILMN_1762003 | *TLOC1* |
| 117 | ILMN_2107184 | *SNRK* |
| 118 | ILMN_1658071 | *ATP1B1* |
| 119 | ILMN_1762531 | *FGF9* |
| 120 | ILMN_1811650 | *DUS2L* |
| 121 | ILMN_1682799 | *STAMBPL1* |
| 122 | ILMN_1752117 | *TMEM55A* |
| 123 | ILMN_1682095 | *RCOR3* |
| 124 | ILMN_1731612 | *UCHL5* |
| 125 | ILMN_2069224 | *PVALB* |
| 126 | ILMN_1721138 | *GRPEL2* |
| 127 | ILMN_1787658 | *MTMR7* |
| 128 | ILMN_1801378 | *COQ3* |
| 129 | ILMN_1683576 | *MAGED2* |
| 130 | ILMN_1697959 | *SLC35B4* |
| 131 | ILMN_1813344 | *C20orf7* |
| 132 | ILMN_1744968 | *KCNAB1* |
| 133 | ILMN_1799128 | *SLC30A9* |
| 134 | ILMN_1772627 | *D4S234E* |
| 135 | ILMN_1732810 | *SNX17* |
| 136 | ILMN_1652540 | *C5orf16* |
| 137 | ILMN_1756701 | *MGC4172* |
| 138 | ILMN_1805481 | *C6orf75* |
| 139 | ILMN_2405470 | *KARCA1* |
| 140 | ILMN_1684271 | *ACBD6* |
| 141 | ILMN_1705153 | *NEFH* |
| 142 | ILMN_1721669 | *IDH3B* |
| 143 | ILMN_2123431 | *RPS6KC1* |
| 144 | ILMN_2079786 | *NUAK1* |
| 145 | ILMN_1657682 | *GFM1* |
| 146 | ILMN_1685496 | *RGS7* |
| 147 | ILMN_1779428 | *LOC387856* |
| 148 | ILMN_1786966 | *ATRNL1* |
| 149 | ILMN_2343048 | *ABCB9* |
| 150 | ILMN_1777745 | *MGC40405* |
| 151 | ILMN_2388975 | *CERK* |
| 152 | ILMN_1798485 | *ATP6V1E1* |
| 153 | ILMN_1742224 | *SLTM* |
| 154 | ILMN_2181191 | *TPI1* |
| 155 | ILMN_2119224 | *KIFAP3* |
| 156 | ILMN_1678692 | *M-RIP* |
| 157 | ILMN_1813091 | *ARL1* |
| 158 | ILMN_1794829 | *C6orf117* |
| 159 | ILMN_1791006 | *AHI1* |
| 160 | ILMN_2075643 | *ANKRD29* |
| 161 | ILMN_1676749 | *C3orf15* |
| 162 | ILMN_2373632 | *IDH3B* |
| 163 | ILMN_2082209 | *C20orf100* |
| 164 | ILMN_2211583 | *KIAA1279* |
| 165 | ILMN_1671494 | *USP5* |
| 166 | ILMN_1793371 | *LKAP* |
| 167 | ILMN_1688515 | *ZNF195* |
| 168 | ILMN_2288070 | *FTO* |
| 169 | ILMN_1767365 | *PAK1* |
| 170 | ILMN_1691436 | *BLVRA* |
| 171 | ILMN_1796069 | *CBLN2* |
| 172 | ILMN_1656300 | *GFRA2* |
| 173 | ILMN_1814787 | *ICA1* |
| 174 | ILMN_2339779 | *ATP6V1E1* |
| 175 | ILMN_1765001 | *NAT6* |
| 176 | ILMN_1763634 | *PEX14* |
| 177 | ILMN_1784630 | *KBTBD11* |
| 178 | ILMN_1660732 | *PPP2R2B* |
| 179 | ILMN_1813746 | *CORO2A* |
| 180 | ILMN_1688188 | *CADPS* |
| 181 | ILMN_2087941 | *ENTPD3* |
| 182 | ILMN_1687279 | *DHPS* |
| 183 | ILMN_1672121 | *LOC387856* |
| 184 | ILMN_1662419 | *COX7A1* |
| 185 | ILMN_2341363 | *ATP5A1* |
| 186 | ILMN_1781151 | *ARMC8* |
| 187 | ILMN_1758825 | *ABLIM2* |
| 188 | ILMN_1746426 | *TOMM70A* |
| 189 | ILMN_1733991 | *UBL7* |
| 190 | ILMN_1666625 | *KIF17* |
| 191 | ILMN_2343624 | *KIAA0859* |
| 192 | ILMN_1790575 | *KIAA0859* |
| 193 | ILMN_1796497 | *PIP3-E* |
| 194 | ILMN_2043809 | *PFKM* |
| 195 | ILMN_1788604 | *WBP2* |
| 196 | ILMN_1695821 | *ACTR1B* |
| 197 | ILMN_1751689 | *CHRM1* |
| 198 | ILMN_2048591 | *LRRN3* |
| 199 | ILMN_2405756 | *VAMP1* |
| 200 | ILMN_1659952 | *MTMR2* |
| 201 | ILMN_2387865 | *KCNIP4* |
| 202 | ILMN_2044226 | *PPP3CA* |
| 203 | ILMN_1750876 | *SLC4A1AP* |
| 204 | ILMN_1653200 | *SLC22A17* |
| 205 | ILMN_1706960 | *KIAA1217* |
| 206 | ILMN_1656840 | *VPS13D* |
| 207 | ILMN_1673704 | *INA* |
| 208 | ILMN_1743747 | *RUSC1* |
| 209 | ILMN_2087528 | *CPSF3* |
| 210 | ILMN_2345319 | *PREPL* |
| 211 | ILMN_1723837 | *SLC6A17* |
| 212 | ILMN_1720158 | *ETS2* |
| 213 | ILMN_1733696 | *IMP3* |
| 214 | ILMN_1710303 | *TTC25* |
| 215 | ILMN_1768488 | *TERF2* |
| 216 | ILMN_1688034 | *COIL* |
| 217 | ILMN_1776104 | *NDUFS5* |
| 218 | ILMN_1796923 | *LOC81691* |
| 219 | ILMN_2202915 | *MLSTD1* |
| 220 | ILMN_1751868 | *TCTEX1D1* |
| 221 | ILMN_1694799 | *PIAS2* |
| 222 | ILMN_2377459 | *PSCD2* |
| 223 | ILMN_1784292 | *ANKMY2* |
| 224 | ILMN_1665761 | *BCL11B* |
| 225 | ILMN_2073592 | *CAND2* |
| 226 | ILMN_1801090 | *KA21* |
| 227 | ILMN_1799890 | *ZFYVE20* |
| 228 | ILMN_1654217 | *MPP2* |
| 229 | ILMN_1705064 | *NDEL1* |
| 230 | ILMN_1703791 | *ANXA7* |
| 231 | ILMN_2412139 | *CABYR* |
| 232 | ILMN_2062754 | *SRA1* |
| 233 | ILMN_1697906 | *WBP4* |
| 234 | ILMN_1657797 | *FIBP* |
| 235 | ILMN_1684040 | *C6orf190* |
| 236 | ILMN_1718972 | *MFSD3* |
| 237 | ILMN_1653670 | *CUL3* |
| 238 | ILMN_1765796 | *ENO2* |
| 239 | ILMN_1673820 | *SMARCA3* |
| 240 | ILMN_1794213 | *ABHD14A* |
| 241 | ILMN_1732555 | *B4GALT6* |
| 242 | ILMN_1737635 | *RAD1* |
| 243 | ILMN_2407824 | *ATP1B1* |
| 244 | ILMN_1658289 | *WDR54* |
| 245 | ILMN_1677439 | *GLS2* |
| 246 | ILMN_1664071 | *TNNT2* |
| 247 | ILMN_1774350 | *MYOZ3* |
| 248 | ILMN_2379520 | *HAGH* |
| 249 | ILMN_1781060 | *SYN2* |
| 250 | ILMN_1804339 | *CAMK1G* |
| 251 | ILMN_1728934 | *PRC1* |
| 252 | ILMN_2395055 | *ATPAF1* |
| 253 | ILMN_1695491 | *C8orf32* |
| 254 | ILMN_2359789 | *RAC1* |
| 255 | ILMN_1714577 | *OGDHL* |
| 256 | ILMN_1810533 | *SLC6A15* |
| 257 | ILMN_1788149 | *NEK11* |
| 258 | ILMN_1668484 | *LRRC47* |
| 259 | ILMN_1653612 | *KCTD9* |
| 260 | ILMN_1722559 | *NEUROD6* |
| 261 | ILMN_1717934 | *SYT11* |
| 262 | ILMN_1801703 | *CPLX1* |
| 263 | ILMN_1658717 | *DEPDC5* |
| 264 | ILMN_1705093 | *ORC5L* |
| 265 | ILMN_1680353 | *NSF* |
| 266 | ILMN_1770623 | *FAM58A* |
| 267 | ILMN_1715832 | *PIK3R4* |
| 268 | ILMN_2051232 | *SDHA* |
| 269 | ILMN_1729816 | *VDAC3* |
| 270 | ILMN_1789040 | *SLITRK5* |
| 271 | ILMN_2343097 | *NCALD* |
| 272 | ILMN_1793615 | *ME3* |
| 273 | ILMN_1753064 | *TTC13* |
| 274 | ILMN_1665730 | *ABCB10* |
| 275 | ILMN_1656145 | *GOT1* |
| 276 | ILMN_2344650 | *CG018* |
| 277 | ILMN_1723886 | *ZFP2* |
| 278 | ILMN_2312709 | *LCMT1* |
| 279 | ILMN_2109197 | *EPB41L3* |
| 280 | ILMN_2182750 | *DDX1* |
| 281 | ILMN_2348243 | *LYK5* |
| 282 | ILMN_1777765 | *C12orf10* |
| 283 | ILMN_1684931 | *GPR119* |
| 284 | ILMN_1669607 | *PHKG2* |
| 285 | ILMN_1813236 | *C6orf136* |
| 286 | ILMN_1783805 | *PNMA3* |
| 287 | ILMN_1701940 | *FAM73B* |
| 288 | ILMN_1706935 | *NAG6* |
| 289 | ILMN_1693401 | *BTBD5* |
| 290 | ILMN_1655418 | *CAPNS1* |
| 291 | ILMN_1737344 | *DDX41* |
| 292 | ILMN_1751431 | *WIBG* |
| 293 | ILMN_2176931 | *PELI3* |
| 294 | ILMN_1745807 | *TMEM62* |
| 295 | ILMN_1774596 | *BSCL2* |
| 296 | ILMN_1742147 | *UBL4A* |
| 297 | ILMN_1793522 | *PRKAB1* |
| 298 | ILMN_2255579 | *RAB37* |
| 299 | ILMN_1713803 | *LOC400566* |
| 300 | ILMN_1669382 | *CPLX2* |
| 301 | ILMN_1712517 | *ZNF696* |
| 302 | ILMN_1797362 | *LIMK1* |
| 303 | ILMN_1706266 | *RNF157* |
| 304 | ILMN_1732772 | *PPME1* |
| 305 | ILMN_1775048 | *DIRAS1* |
| 306 | ILMN_1740772 | *APBB3* |
| 307 | ILMN_2371379 | *ACLY* |
| 308 | ILMN_1706057 | *CS* |
| 309 | ILMN_1780698 | *ZFYVE19* |
| 310 | ILMN_2341467 | *PARL* |
| 311 | ILMN_1675709 | *ARFGAP1* |
| 312 | ILMN_1715636 | *EIF3S9* |
| 313 | ILMN_1721575 | *VPS18* |
| 314 | ILMN_1725707 | *ATG16L1* |
| 315 | ILMN_1759613 | *PNMA6A* |
| 316 | ILMN_1700461 | *AARSD1* |
| 317 | ILMN_2366634 | *PKM2* |
| 318 | ILMN_1763523 | *HARS* |
| 319 | ILMN_1769545 | *PRPF19* |
| 320 | ILMN_1794875 | *HMFN0839* |
| 321 | ILMN_1708059 | *USP13* |
| 322 | ILMN_1720482 | *BM88* |
| 323 | ILMN_1729748 | *PEF1* |
| 324 | ILMN_1735743 | *FLJ37440* |
| 325 | ILMN_1761828 | *E2F4* |
| 326 | ILMN_1737847 | *FLJ22318* |
| 327 | ILMN_1666076 | *ARHGEF4* |
| 328 | ILMN_1719998 | *C9orf45* |
| 329 | ILMN_1663032 | *FNDC4* |
| 330 | ILMN_1811560 | *SF4* |
| 331 | ILMN_2308689 | *FLJ21839* |
| 332 | ILMN_1653599 | *ATP5D* |
| 333 | ILMN_1804789 | *KIAA1967* |
| 334 | ILMN_1792168 | *GALE* |
| 335 | ILMN_1690523 | *LRRC20* |
| 336 | ILMN_1659086 | *NEFL* |
| 337 | ILMN_1749210 | *MGC13125* |
| 338 | ILMN_1774604 | *PNKD* |
| 339 | ILMN_1751571 | *RAD23A* |
| 340 | ILMN_2366710 | *UCRC* |
| 341 | ILMN_1781516 | *SUPT16H* |
| 342 | ILMN_1729075 | *PTHR2* |
| 343 | ILMN_1724774 | *SYT3* |
| 344 | ILMN_1798620 | *PQLC1* |
| 345 | ILMN_1743836 | *MXRA7* |
| 346 | ILMN_1665526 | *TCEA2* |
| 347 | ILMN_1705364 | *BAT3* |
| 348 | ILMN_1802706 | *IDH3G* |
| 349 | ILMN_1810467 | *PPP2R1A* |
| 350 | ILMN_2320513 | *APBB3* |
| 351 | ILMN_1676393 | *ATP5G1* |
| 352 | ILMN_1802646 | *EPHB6* |
| 353 | ILMN_1808299 | *IQSEC1* |
| 354 | ILMN_2397842 | *SNCB* |
| 355 | ILMN_1784320 | *ELMO1* |
| 356 | ILMN_1661170 | *NDUFB8* |
| 357 | ILMN_1815682 | *C3orf37* |
| 358 | ILMN_2415189 | *ATP1A1* |
| 359 | ILMN_1733861 | *ARHGEF11* |
| 360 | ILMN_1695947 | *SCN4B* |
| 361 | ILMN_1657554 | *TSPYL2* |
| 362 | ILMN_1779064 | *KLC2* |
| 363 | ILMN_1753345 | *SCAMP5* |
| 364 | ILMN_1746579 | *UBE2O* |
| 365 | ILMN_1798458 | *KIAA1026* |
| 366 | ILMN_2403555 | *DHX30* |
| 367 | ILMN_1652486 | *THAP7* |
| 368 | ILMN_1718295 | *STAC2* |
| 369 | ILMN_1697694 | *ATP6AP1* |
| 370 | ILMN_1767256 | *MBTPS1* |
| 371 | ILMN_1711208 | *CELSR2* |
| 372 | ILMN_1740604 | *RAB11FIP5* |
| 373 | ILMN_1718783 | *EXTL1* |

## Supplementary Table S3. The 31 terms of biological processes enriched by the differentially expressed genes of ASD.

| **GO number** | **Term** | **Count** | **P-Value** |
| --- | --- | --- | --- |
| GO:0006099 | tricarboxylic acid cycle | 7 | 5.22E-06 |
| GO:0006836 | neurotransmitter transport | 5 | 6.99E-04 |
| GO:0015991 | ATP hydrolysis coupled proton transport | 5 | 0.001561326 |
| GO:0016079 | synaptic vesicle exocytosis | 4 | 0.004843637 |
| GO:0061621 | canonical glycolysis | 4 | 0.007805819 |
| GO:0006887 | exocytosis | 6 | 0.010230961 |
| GO:0051592 | response to calcium ion | 5 | 0.013378217 |
| GO:0006096 | glycolytic process | 4 | 0.016376575 |
| GO:0030036 | actin cytoskeleton organization | 7 | 0.017451124 |
| GO:0072659 | protein localization to plasma membrane | 5 | 0.017671671 |
| GO:0008610 | lipid biosynthetic process | 3 | 0.020066132 |
| GO:1901379 | regulation of potassium ion transmembrane transport | 3 | 0.022914303 |
| GO:0051262 | protein tetramerization | 4 | 0.025235052 |
| GO:0000278 | mitotic cell cycle | 4 | 0.026916709 |
| GO:0006468 | protein phosphorylation | 14 | 0.030259284 |
| GO:0035552 | oxidative single-stranded DNA demethylation | 2 | 0.031431748 |
| GO:1990504 | dense core granule exocytosis | 2 | 0.031431748 |
| GO:0015820 | leucine transport | 2 | 0.031431748 |
| GO:0006094 | gluconeogenesis | 4 | 0.032311874 |
| GO:0060048 | cardiac muscle contraction | 4 | 0.034226308 |
| GO:0015992 | proton transport | 4 | 0.042456454 |
| GO:0045921 | positive regulation of exocytosis | 3 | 0.043075925 |
| GO:0042776 | mitochondrial ATP synthesis coupled proton transport | 3 | 0.043075925 |
| GO:0035553 | oxidative single-stranded RNA demethylation | 2 | 0.046776546 |
| GO:0035494 | SNARE complex disassembly | 2 | 0.046776546 |
| GO:0033693 | neurofilament bundle assembly | 2 | 0.046776546 |
| GO:0045903 | positive regulation of translational fidelity | 2 | 0.046776546 |
| GO:0006419 | alanyl-tRNA aminoacylation | 2 | 0.046776546 |
| GO:0009451 | RNA modification | 2 | 0.046776546 |
| GO:0015986 | ATP synthesis coupled proton transport | 3 | 0.046899154 |
| GO:1901796 | regulation of signal transduction by p53 class mediator | 6 | 0.047423722 |

## Supplementary Table S4. The 19 terms of cellular components enriched by the differentially expressed genes of ASD.

| **GO number** | **Term** | **Count** | **P-Value** |
| --- | --- | --- | --- |
| GO:0005739 | mitochondrion | 46 | 4.4777E-07 |
| GO:0043209 | myelin sheath | 14 | 6.57913E-07 |
| GO:0005829 | cytosol | 85 | 8.91298E-07 |
| GO:0005774 | vacuolar membrane | 5 | 4.83559E-05 |
| GO:0030672 | synaptic vesicle membrane | 6 | 0.001534728 |
| GO:0005759 | mitochondrial matrix | 14 | 0.001712366 |
| GO:0016020 | membrane | 51 | 0.00286294 |
| GO:0005883 | neurofilament | 3 | 0.00619586 |
| GO:0014069 | postsynaptic density | 9 | 0.007707566 |
| GO:0008021 | synaptic vesicle | 6 | 0.013736852 |
| GO:0098793 | presynapse | 5 | 0.016859185 |
| GO:0015630 | microtubule cytoskeleton | 7 | 0.019278115 |
| GO:0005730 | nucleolus | 22 | 0.02411259 |
| GO:0070554 | synaptobrevin 2-SNAP-25-syntaxin-3-complexin complex | 2 | 0.030493476 |
| GO:0014704 | intercalated disc | 4 | 0.031696666 |
| GO:0005743 | mitochondrial inner membrane | 13 | 0.040450188 |
| GO:0030667 | secretory granule membrane | 3 | 0.040768378 |
| GO:0005753 | mitochondrial proton-transporting ATP synthase complex | 3 | 0.040768378 |
| GO:0031201 | SNARE complex | 4 | 0.047895725 |

## Supplementary Table S5. The 22 terms of molecular functions enriched by the differentially expressed genes of ASD.

| **GO number** | **Term** | **Count** | **P-Value** |
| --- | --- | --- | --- |
| GO:0005524 | ATP binding | 44 | 0.0002032 |
| GO:0005515 | protein binding | 172 | 0.00066074 |
| GO:0004449 | isocitrate dehydrogenase (NAD+) activity | 3 | 0.00079615 |
| GO:0046933 | proton-transporting ATP synthase activity, rotational mechanism | 4 | 0.00250618 |
| GO:0004672 | protein kinase activity | 14 | 0.00654225 |
| GO:0046961 | proton-transporting ATPase activity, rotational mechanism | 4 | 0.00859575 |
| GO:0019894 | kinesin binding | 4 | 0.01942614 |
| GO:0019901 | protein kinase binding | 13 | 0.02188139 |
| GO:0051287 | NAD binding | 4 | 0.02415926 |
| GO:0005509 | calcium ion binding | 20 | 0.02756881 |
| GO:0008022 | protein C-terminus binding | 8 | 0.0307301 |
| GO:0016616 | oxidoreductase activity, acting on the CH-OH group of donors, NAD or NADP as acceptor | 3 | 0.03102309 |
| GO:0017075 | syntaxin-1 binding | 3 | 0.03452958 |
| GO:0005200 | structural constituent of cytoskeleton | 6 | 0.03493832 |
| GO:0019905 | syntaxin binding | 5 | 0.03780247 |
| GO:0004527 | exonuclease activity | 3 | 0.03818178 |
| GO:0008289 | lipid binding | 7 | 0.03846523 |
| GO:0004843 | thiol-dependent ubiquitin-specific protease activity | 5 | 0.04255622 |
| GO:0008601 | protein phosphatase type 2A regulator activity | 3 | 0.04590073 |
| GO:0004674 | protein serine/threonine kinase activity | 12 | 0.04676784 |
| GO:0003872 | 6-phosphofructokinase activity | 2 | 0.04842642 |
| GO:0004416 | hydroxyacylglutathione hydrolase activity | 2 | 0.04842642 |

## Supplementary Table S6. The pathways associated with differentially expressed genes were enriched by using DAVID.

| **Category** | **Term** | **Count** | **%** | **P-Value** |
| --- | --- | --- | --- | --- |
| KEGG_PATHWAY | hsa01200:Carbon metabolism | 13 | 4.40678 | 1.86E-07 |
| KEGG_PATHWAY | hsa00020:Citrate cycle (TCA cycle) | 8 | 2.711864 | 3.22E-07 |
| KEGG_PATHWAY | hsa01230:Biosynthesis of amino acids | 10 | 3.389831 | 2.20E-06 |
| KEGG_PATHWAY | hsa01210:2-Oxocarboxylic acid metabolism | 5 | 1.694915 | 1.23E-04 |
| KEGG_PATHWAY | hsa01130:Biosynthesis of antibiotics | 13 | 4.40678 | 1.27E-04 |
| KEGG_PATHWAY | hsa00190:Oxidative phosphorylation | 10 | 3.389831 | 2.48E-04 |
| KEGG_PATHWAY | hsa05012:Parkinson's disease | 9 | 3.050847 | 0.00183213 |
| KEGG_PATHWAY | hsa01100:Metabolic pathways | 33 | 11.18644 | 0.00223826 |
| KEGG_PATHWAY | hsa04932:Non-alcoholic fatty liver disease (NAFLD) | 9 | 3.050847 | 0.00269508 |
| KEGG_PATHWAY | hsa05010:Alzheimer's disease | 9 | 3.050847 | 0.00516564 |
| KEGG_PATHWAY | hsa04260:Cardiac muscle contraction | 6 | 2.033898 | 0.00665479 |
| KEGG_PATHWAY | hsa05016:Huntington's disease | 9 | 3.050847 | 0.01123216 |
| KEGG_PATHWAY | hsa05120:Epithelial cell signaling in Helicobacter pylori infection | 5 | 1.694915 | 0.02163265 |
| BIOCARTA | h_etcPathway:Electron Transport Reaction in Mitochondria | 3 | 1.016949 | 0.02564577 |
| KEGG_PATHWAY | hsa04261:Adrenergic signaling in cardiomyocytes | 7 | 2.372881 | 0.02857479 |
| KEGG_PATHWAY | hsa03018:RNA degradation | 5 | 1.694915 | 0.03388427 |
| KEGG_PATHWAY | hsa04152:AMPK signaling pathway | 6 | 2.033898 | 0.04463936 |
| KEGG_PATHWAY | hsa05014:Amyotrophic lateral sclerosis (ALS) | 4 | 1.355932 | 0.04483325 |

## Supplementary Table S7. The biological processes (BP) and cellular components (CC) associated with differentially expressed genes were analyzed by using ClueGO.

| **GO ID** | **GO Term** | **Term**  **P-Value** | **Group**  **P-Value** | **GO Groups*** |
| --- | --- | --- | --- | --- |
| GO:0033178 | proton-transporting two-sector ATPase complex, catalytic domain | 0.00 | 0.00 | Group0 |
| GO:0019320 | hexose catabolic process | 0.00 | 0.00 | Group1 |
| GO:0006007 | glucose catabolic process | 0.00 | 0.00 | Group1 |
| GO:0098685 | Schaffer collateral - CA1 synapse | 0.00 | 0.00 | Group2 |
| GO:0060052 | neurofilament cytoskeleton organization | 0.00 | 0.00 | Group2 |
| GO:0099186 | structural constituent of postsynapse | 0.00 | 0.00 | Group2 |
| GO:0099185 | postsynaptic intermediate filament cytoskeleton organization | 0.00 | 0.00 | Group2 |
| GO:0099184 | structural constituent of postsynaptic intermediate filament cytoskeleton | 0.00 | 0.00 | Group2 |
| GO:0006091 | generation of precursor metabolites and energy | 0.00 | 0.00 | Group3 |
| GO:0015980 | energy derivation by oxidation of organic compounds | 0.00 | 0.00 | Group3 |
| GO:0045333 | cellular respiration | 0.00 | 0.00 | Group3 |
| GO:0006099 | tricarboxylic acid cycle | 0.00 | 0.00 | Group3 |
| GO:0009060 | aerobic respiration | 0.00 | 0.00 | Group3 |
| GO:0004448 | isocitrate dehydrogenase activity | 0.00 | 0.00 | Group3 |
| GO:0006102 | isocitrate metabolic process | 0.00 | 0.00 | Group3 |
| GO:0072350 | tricarboxylic acid metabolic process | 0.00 | 0.00 | Group3 |
| GO:0004449 | isocitrate dehydrogenase (NAD+) activity | 0.00 | 0.00 | Group3 |
| GO:0006836 | neurotransmitter transport | 0.00 | 0.00 | Group4 |
| GO:0060627 | regulation of vesicle-mediated transport | 0.00 | 0.00 | Group4 |
| GO:0098978 | glutamatergic synapse | 0.00 | 0.00 | Group4 |
| GO:0099003 | vesicle-mediated transport in synapse | 0.00 | 0.00 | Group4 |
| GO:0099504 | synaptic vesicle cycle | 0.00 | 0.00 | Group4 |
| GO:0099643 | signal release from synapse | 0.00 | 0.00 | Group4 |
| GO:0017157 | regulation of exocytosis | 0.00 | 0.00 | Group4 |
| GO:0007269 | neurotransmitter secretion | 0.00 | 0.00 | Group4 |
| GO:0016079 | synaptic vesicle exocytosis | 0.00 | 0.00 | Group4 |
| GO:0017156 | calcium-ion regulated exocytosis | 0.00 | 0.00 | Group4 |
| GO:0046928 | regulation of neurotransmitter secretion | 0.00 | 0.00 | Group4 |

*The different GO Groups are indicated with different color corresponding to Figure 3A.

## Supplementary Table S8. The pathways associated with differentially expressed genes were analyzed by using ClueGO.

| **GOID** | **GOTerm** | **Term** PValue | **Group** PValue | **GOGroups*** |
| --- | --- | --- | --- | --- |
| R-HSA:1268020 | Mitochondrial protein import | 0.00 | 0.00 | Group0 |
| R-HSA:9609507 | Protein localization | 0.00 | 0.00 | Group0 |
| R-HSA:70326 | Glucose metabolism | 0.00 | 0.00 | Group1 |
| WP:534 | Glycolysis and Gluconeogenesis | 0.00 | 0.00 | Group1 |
| KEGG:00020 | Citrate cycle (TCA cycle) | 0.00 | 0.00 | Group2 |
| KEGG:00190 | Oxidative phosphorylation | 0.00 | 0.00 | Group2 |
| KEGG:04714 | Thermogenesis | 0.00 | 0.00 | Group2 |
| KEGG:04932 | Non-alcoholic fatty liver disease (NAFLD) | 0.00 | 0.00 | Group2 |
| KEGG:05012 | Parkinson disease | 0.00 | 0.00 | Group2 |
| KEGG:05016 | Huntington disease | 0.00 | 0.00 | Group2 |
| R-HSA:1428517 | The citric acid (TCA) cycle and respiratory electron transport | 0.00 | 0.00 | Group2 |
| R-HSA:163200 | Respiratory electron transport, ATP synthesis by chemiosmotic coupling, and heat production by uncoupling proteins. | 0.00 | 0.00 | Group2 |
| R-HSA:71403 | Citric acid cycle (TCA cycle) | 0.00 | 0.00 | Group2 |
| R-HSA:71406 | Pyruvate metabolism and Citric Acid (TCA) cycle | 0.00 | 0.00 | Group2 |
| WP:111 | Electron Transport Chain (OXPHOS system in mitochondria) | 0.00 | 0.00 | Group2 |
| WP:2453 | TCA Cycle and Deficiency of Pyruvate Dehydrogenase complex (PDHc) | 0.00 | 0.00 | Group2 |
| WP:78 | TCA Cycle (aka Krebs or citric acid cycle) | 0.00 | 0.00 | Group2 |

*The different GO Groups are indicated with different color corresponding to Figure 3B.

## Supplementary Table S9. The 59 proteins predicted to be ASD-related in blood.

| **No.** | **Gene Symbol** | **UniProt ID** | **Protein** |
| --- | --- | --- | --- |
| 1 | *AARS* | P49588 | Alanine--tRNA ligase, cytoplasmic |
| 2 | *ACLY* | P53396 | ATP-citrate synthase |
| 3 | *ACTL6B* | O94805 | Actin-like protein 6B |
| 4 | *ACTR1B* | P42025 | Beta-centractin |
| 5 | *ARHGEF4* | Q9NR80 | Rho guanine nucleotide exchange factor 4 |
| 6 | *ARMC8* | Q8IUR7 | Armadillo repeat-containing protein 8 |
| 7 | ***ATPAF1****^*,§^* | Q5TC12 | ATP synthase mitochondrial F1 complex assembly factor 1 |
| 8 | *CDK5RAP1* | Q96SZ6 | CDK5 regulatory subunit-associated protein 1 |
| 9 | *CERK* | Q8TCT0 | Ceramide kinase |
| 10 | *COPG* | Q9Y678 | Coatomer subunit gamma-1 |
| 11 | *CPSF3* | Q9UKF6 | Cleavage and polyadenylation specificity factor subunit 3 |
| 12 | *DDX1* | Q92499 | ATP-dependent RNA helicase DDX1 |
| 13 | *DHX35* | Q9H5Z1 | Probable ATP-dependent RNA helicase DHX35 |
| 14 | *DPP8* | Q6V1X1 | Dipeptidyl peptidase 8 |
| 15 | *EPHB6* | O15197 | Ephrin type-B receptor 6 |
| 16 | *EPS15* | P42566 | Epidermal growth factor receptor substrate 15 |
| 17 | *ETS2* | P15036 | Protein C-ets-2 |
| 18 | *EXOC3(SEC6L1)* | O60645 | Exocyst complex component 3 |
| 19 | *EXTL1* | Q92935 | Exostosin-like 1 |
| 20 | *FTO* | Q9C0B1 | Alpha-ketoglutarate-dependent dioxygenase FTO |
| 21 | *GFM1* | Q96RP9 | Elongation factor G, mitochondrial |
| 22 | *GFRA2* | O00451 | GDNF family receptor alpha-2 |
| 23 | *GLMN* | Q92990 | Glomulin |
| 24 | *KCTD9* | Q7L273 | BTB/POZ domain-containing protein KCTD9 |
| 25 | *LIMK1* | P53667 | LIM domain kinase 1 |
| 26 | *MAPK10* | P53779 | Mitogen-activated protein kinase 10 |
| 27 | *METTL13(KIAA0859)* | Q8N6R0 | Methyltransferase-like protein 13 |
| 28 | *NEPRO(C3orf17)* | Q6NW34 | Nucleolus and neural progenitor protein |
| 29 | *NFXL1* | Q6ZNB6 | NF-X1-type zinc finger protein NFXL1 |
| 30 | *PAM* | P19021 | Peptidyl-glycine alpha-amidating monooxygenase |
| 31 | *PCSK1* | P29120 | Neuroendocrine convertase 1 |
| 32 | *PFKM* | P08237 | ATP-dependent 6-phosphofructokinase, muscle type |
| 33 | *PGBD1* | Q96JS3 | PiggyBac transposable element-derived protein 1 |
| 34 | *PIK3R4* | Q99570 | Phosphoinositide 3-kinase regulatory subunit 4 |
| 35 | *POLR3C* | Q9BUI4 | DNA-directed RNA polymerase III subunit RPC3 |
| 36 | *PPP2R1A* | P30153 | Serine/threonine-protein phosphatase 2A 65 kDa regulatory subunit A alpha isoform |
| 37 | *PPP2R2B* | Q00005 | Serine/threonine-protein phosphatase 2A 55 kDa regulatory subunit B beta isoform |
| 38 | *PPP3CA* | Q08209 | Serine/threonine-protein phosphatase 2B catalytic subunit alpha isoform |
| 39 | *PREPL* | Q4J6C6 | Prolyl endopeptidase-like |
| 40 | *PRKAA1* | Q13131 | 5'-AMP-activated protein kinase catalytic subunit alpha-1 |
| 41 | *RAD1* | O60671 | Cell cycle checkpoint protein RAD1 |
| 42 | ***RARS*** | P54136 | Arginine--tRNA ligase, cytoplasmic |
| 43 | *RBSN(ZFYVE20)* | Q9H1K0 | Rabenosyn-5 |
| 44 | *RGS7* | P49802 | Regulator of G-protein signaling 7 |
| 45 | *RNF157* | Q96PX1 | E3 ubiquitin ligase RNF157 |
| 46 | *SLC25A12* | O75746 | Calcium-binding mitochondrial carrier protein Aralar1 |
| 47 | *SLC25A27* | O95847 | Mitochondrial uncoupling protein 4 |
| 48 | *SLITRK5* | O94991 | SLIT and NTRK-like protein 5 |
| 49 | *STAC2* | Q6ZMT1 | SH3 and cysteine-rich domain-containing protein 2 |
| 50 | *STAMBPL1* | Q96FJ0 | AMSH-like protease |
| 51 | *STAU2* | Q9NUL3 | Double-stranded RNA-binding protein Staufen homolog 2 |
| 52 | *SYT13* | Q7L8C5 | Synaptotagmin-13 |
| 53 | ***THOC5*** | Q13769 | THO complex subunit 5 homolog |
| 54 | *TPK1* | Q9H3S4 | Thiamin pyrophosphokinase 1 |
| 55 | *TRMT11(C6orf75)* | Q7Z4G4 | tRNA (guanine(10)-N2)-methyltransferase homolog |
| 56 | ***TTC13*** | Q8NBP0 | Tetratricopeptide repeat protein 13 |
| 57 | *TUBGCP5* | Q96RT8 | Gamma-tubulin complex component 5 |
| 58 | *VPS18* | Q9P253 | Vacuolar protein sorting-associated protein 18 homolog |
| 59 | *VRK1* | Q99986 | Serine/threonine-protein kinase VRK1 |

* Bold indicates that these proteins do not match the AutismKB database.

§ Underline indicates that these proteins do not match the plasma protein database.
